# Intensive care unit-acquired *Stenotrophomonas maltophilia*: incidence, risk factors, and outcome

**DOI:** 10.1186/cc5063

**Published:** 2006-10-06

**Authors:** Saad Nseir, Christophe Di Pompeo, Hélène Brisson, Florent Dewavrin, Stéphanie Tissier, Maimouna Diarra, Marie Boulo, Alain Durocher

**Affiliations:** 1 Intensive Care Unit, Calmette Hospital, University Hospital of Lille, boulevard du Pr Leclercq, 59037 Lille cedex, France; 2 Medical Assessment Laboratory, Lille II University, 1 place de Verdun, 59045 Lille, France

## Abstract

**Introduction:**

The aim of this study was to determine incidence, risk factors, and impact on outcome of intensive care unit (ICU)-acquired *Stenotrophomonas maltophilia*.

**Methods:**

This prospective observational case-control study, which was a part of a cohort study, was conducted in a 30-bed ICU during a three year period. All immunocompetent patients hospitalised >48 hours were eligible. Patients with non-fermenting Gram-negative bacilli (NF-GNB) at ICU admission were excluded. Patients without ICU-acquired *S. maltophilia *who developed an ICU-acquired NF-GNB other than *S. maltophilia *were also excluded. Screening (tracheal aspirate and skin, anal, and nasal swabs) for NF-GNB was performed in all patients at ICU admission and weekly. Univariate and multivariate analyses were performed to determine risk factors for ICU-acquired *S. maltophilia *and for ICU mortality.

**Results:**

Thirty-eight (2%) patients developed an *S. maltophilia *ICU-acquired colonisation and/or infection and were all successfully matched with 76 controls. Chronic obstructive pulmonary disease (COPD) and duration of antibiotic treatment (odds ratio [OR] [95% confidence interval (CI)] = 9.4 [3 to 29], *p *< 0.001, and 1.4 [1 to 2.3], *p *= 0.001, respectively) were independently associated with ICU-acquired *S. maltophilia*. Mortality rate (60% versus 40%, OR [95% CI] = 1.3 [1 to 1.7, *p *= 0.037]), duration of mechanical ventilation (23 ± 16 versus 7 ± 11 days, *p *< 0.001), and duration of ICU stay (29 ± 21 versus 15 ± 17 days, *p *< 0.001) were significantly higher in cases than in controls. In addition, ICU-acquired infection related to *S. maltophilia *was independently associated with ICU mortality (OR [95% CI] = 2.8 [1 to 7.7], *p *= 0.044).

**Conclusion:**

COPD and duration of antibiotic treatment are independent risk factors for ICU-acquired *S. maltophilia*. ICU-acquired *S. maltophilia *is associated with increased morbidity and mortality rates. ICU-acquired infection related to *S. maltophilia *is an independent risk factor for ICU mortality.

## Introduction

Non-fermenting Gram-negative bacilli (NF-GNB) colonisation and infection are frequent among critically ill patients [1-3]. Antibiotic resistance is common in NF-GNB, resulting in inappropriate initial antibiotic treatment and poor outcomes [4-6]. Several studies have investigated incidence, risk factors, and outcome of patients with *Pseudomonas aeruginosa *and *Acinetobacter baumannii *colonisation and infection. However, few studies have investigated patients with colonisation and/or infection related to *Stenotrophomonas maltophilia*.

Isolation rates of *S. maltophilia *have been increasing since the early 1970s, according to reports from several centres [7]. This pathogen primarily affects patients with co-morbid illness such as cystic fibrosis, immunosuppression, organ transplantation, and malignancies [8]. Infections related to *S. maltophilia *are associated with high morbidity and mortality rates. Therapy for these infections represents a significant challenge both for the clinician and the microbiologist because of this organism's high level of antibiotic resistance to most of the currently used agents and methodological difficulties in susceptibility testing with this organism [9,10].

In recent years, several trials have elucidated risk factors for *S. maltophilia *infection, which include neutropenia, presence of a central venous catheter, prolonged hospitalisation, and previous antibiotic treatment with broad-spectrum antibiotics [11-16]. However, only one study was performed in intensive care unit (ICU) patients with ventilator-associated pneumonia (VAP) related to *S. maltophilia *[16]. In addition, no study has evaluated risk factors for and impact on outcome of ICU-acquired *S. maltophilia*. Identifying risk factors for *S. maltophilia *colonisation and/or infection could be useful for future interventional studies aiming at preventing ICU-acquired *S. maltophilia*. Therefore, we conducted this study to determine incidence of, risk factors for, and outcome of ICU-acquired *S. maltophilia*.

## Materials and methods

This prospective case-control study, which was a part of a cohort study, was conducted in a 30-bed medical and surgical ICU from January 1998 to January 2001. Because the study was observational, Institutional Review Board approval was not required; this was in accordance with Institutional Review Board regulation.

All patients who were without severe immunosuppression and who were hospitalised more than 48 hours in the ICU were eligible. Patients with colonisation and/or infection related to NF-GNB at ICU admission and patients without screening for NF-GNB colonisation at ICU admission and more than 48 hours after ICU admission were excluded. Patients without ICU-acquired *S. maltophilia *who developed an ICU-acquired colonisation and/or infection related to NF-GNB other than *S. maltophilia *were also excluded. However, patients with ICU-acquired colonisation and/or infection related to *S. maltophilia *and ICU-acquired colonisation and/or infection related to NF-GNB other than *S. maltophilia *were studied as cases.

Infection control policy included continuous surveillance of nosocomial infections, isolation techniques, and routine screening of multi-drug-resistant (MDR) bacteria. At ICU admission, isolation techniques were used in all patients until receipt of screening results. Thereafter, these techniques were performed in all patients with colonisation or infection related to MDR bacteria. Isolation techniques included use of protective gowns and gloves and adequate hand-washing with antiseptic soap between patient contacts. No selective digestive decontamination was performed. Antibiotic treatment was based on local antibiotic guidelines, including specific recommendations for each type of infection.

Routine screening of NF-GNB was performed in all patients at ICU admission and on a weekly basis (every Monday) thereafter. This screening included nasal, anal, and axilla swabs. In addition, tracheal aspirate was performed in intubated or tracheotomised patients. Hemocultures, quantitative tracheal aspirates, broncho-alveolar lavage, urine cultures, and other microbiologic cultures were performed according to clinical status.

Infection and colonisation were considered to be ICU-acquired if they were diagnosed more than 48 hours after ICU admission. VAP was defined by the presence of new or progressive radiographic infiltrate associated with two of the following criteria: temperature above 38.5°C or below 36.5°C, leucocyte count above 10,000/μl or below 1,500/μl, purulent tracheal aspirate, and positive (≥10^6 ^colony-forming units per ml) tracheal aspirate. Ventilator-associated tracheobronchitis was defined using all of the following criteria: fever (>38°C) with no other recognisable cause, new or increased sputum production, positive (≥10^6 ^colony-forming units per ml) endotracheal aspirate culture yielding a new bacteria, and no radiographic evidence of new pneumonia [17]. Other definitions of nosocomial infections were based on criteria of the CDC (Centers for Disease Control and Prevention) [18]. Colonisation was defined as a positive microbiologic culture without clinical signs of infection. Prior antibiotic treatment was defined as any antibiotic treatment during the two weeks preceding ICU admission. Severe immunosuppression was defined by the presence of neutropenia (leucocyte count <1,000/μl or neutrophils <500/μl), active solid or hematology malignancy, long-term corticosteroid therapy (≥1 mg/kg per day for >1 month), or HIV infection (CD4 <50/μl during the previous 6 months). Cases were patients with ICU-acquired *S. maltophilia *colonisation and/or infection. Controls were patients without NF-GNB colonisation and/or infection. Antibiotics used during the study period included glycopeptides (vancomycin and teicoplanin), extended-spectrum penicillins (amoxicillin/clavulanate, ticarcillin, ticarcillin/clavulanate, piperacillin, piperacillin/tazobactam, and imipenem), fluoroquinolones (ofloxacin and ciprofloxacin), extended-spectrum cephalosporins (cefotaxime, ceftriaxone, ceftazidime, and cefepime), aminoglycosides (gentamicin, tobramycin, and amikacin), and others (erythromycin, metronidazole, fusidic acid, rifampicin, and fosfomycin). Antimicrobial therapy was deemed inappropriate when none of the antibiotics used was active *in vitro *on *S. maltophilia*. Outcomes evaluated included ICU mortality, duration of mechanical ventilation, and duration of ICU stay.

### Statistical methods

SPSS software (SPSS Inc., Chicago, IL, USA) was used for data analysis. Proportions were compared using the χ^2 ^test or the Fisher exact test whereas appropriate, continuous variables were compared using the Student *t *test or Mann-Whitney *U *test. Results are presented as number (percentage) for frequency and as mean ± standard deviation for quantitative variables. Odds ratio (OR) [95% confidence interval (CI)] was calculated for all significant (*p *< 0.05) qualitative variables in univariate analysis and for all significant variables in multivariate analysis.

Every case patient was matched to two control patients according to all the following criteria: (a) duration of ICU stay before ICU-acquired *S. maltophilia *occurrence (controls ≥ cases), (b) Simplified Acute Physiology Score (SAPS) II at ICU admission ± 5 points, (c) age ± 5 years, (d) admission category (medical/surgical), and (e) date of ICU admission when more than two control patients were well matched to a case. The controls chosen were the ones with the closest date of admission to that of case patients.

Univariate and multivariate analyses were used to determine variables associated with ICU-acquired *S. maltophilia*. The following variables were included in univariate analysis: age, gender, SAPS II and organ failures on ICU admission, transfer from other wards, admission category (medical/surgical), diabetes mellitus, chronic obstructive pulmonary disease (COPD) [19], prior antibiotic treatment, central venous catheter, urinary catheter, tracheostomy, mechanical ventilation, duration of mechanical ventilation, duration of ICU stay, antibiotic treatment, and duration of antibiotic treatment. Treatment with the following antibiotics and its duration were also included in univariate analysis: glycopeptide, extended-spectrum penicillin, fluoroquinolones, extended-spectrum cephalosporin, carbapenem, aminoglycoside, and metronidazole. In patients with ICU-acquired *S. maltophilia*, only exposure to potential risk factors before *S. maltophilia *acquisition was taken into account.

To determine risk factors for ICU-acquired infection related to *S. maltophilia*, cases with only colonisation and their controls were excluded from risk factor analysis. Cases with an ICU-acquired infection and their controls were compared using univariate and multivariate analyses. All the above-cited variables were included in these analyses.

Univariate and multivariate analyses were performed to determine risk factors for ICU mortality among cases and controls. All the above-cited variables were included in univariate analysis. In addition, ICU-acquired *S. maltophilia *and ICU-acquired infection related to *S. maltophilia *were included in risk factor analyses of ICU mortality. Variables with *p *< 0.2 by univariate analysis were included in stepwise logistic regression models. Prior antibiotic use as well as antibiotic treatment in the ICU and its duration were compared between patients with COPD and patients without COPD.

## Results

### Patient characteristics

**Figure 1:**
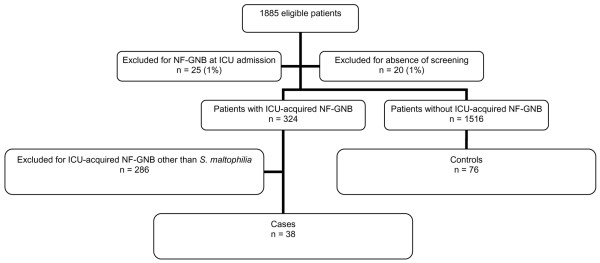
Profile of the study in this report. ICU, intensive care unit; NF-GNB, non-fermenting Gram-negative bacilli

One thousand eight hundred and eighty-five patients were eligible, 25 (1%) patients were excluded for NF-GNB colonisation and/or infection at ICU admission, and 20 (1%) patients for absence of NF-GNB screening at ICU admission and more than 48 hours after ICU admission. Among the 1,840 remaining patients, 324 (17%) patients developed an ICU-acquired NF-GNB. Two hundred and eighty-six (15%) patients without ICU-acquired *S. maltophilia *were excluded for ICU-acquired colonisation and/or infection related to NF-GNB other than *S. maltophilia*. Thirty-eight (2%) patients developed an ICU-acquired colonisation and/or infection related to *S. maltophilia*, representing two patients with ICU-acquired *S. maltophilia *per 1,000 ICU days. These patients were all successfully matched with 76 control patients (Figure 1). Among cases, three patients had an ICU-acquired colonisation and/or infection related to NF-GNB other than *S. maltophilia*. Among cases with ICU-acquired infection related to *S. maltophilia*, 24 (80%) patients had prior colonisation related to *S. maltophilia*.

The mean time between ICU admission and first ICU-acquired *S. maltophilia *was 14 ± 11 days. Thirty of 38 (78%) patients developed 36 ICU-acquired infections related to *S. maltophilia*, including 22 VAP, eight ventilator-associated tracheobronchitis, four urinary infections, and two bacteremias. Patient characteristics are presented in Table 1.

### Risk factors for ICU-acquired *S. maltophilia*

Risk factors for ICU-acquired *S. maltophilia *and for ICU-acquired infection related to *S. maltophilia *which were determined by univariate analysis are presented in Tables 1 and 2. COPD (OR [95% CI] = 9.4 [3 to 29], *p *< 0.001) and duration of antibiotic treatment (OR [95% CI] = 1.4 per day [1 to 2.3], *p *= 0.001) were independently associated with ICU-acquired *S. maltophilia*. Antecedent COPD and duration of antibiotic treatment were also independently associated with ICU-acquired infection related to *S. maltophilia *(OR [95% CI] = 9.1 [2.5 to 32], *p *< 0.001, and 1.16 [1 to 1.2], *p *= 0.001, respectively).

Rates of prior antibiotic use (21 of 41 [51%] versus 27 of 73 [36%], *p *= 0.101) and antibiotic use during ICU stay (32 of 41 [78%] versus 53 of 73 [72%], *p *= 0.342) were similar in patients with COPD as compared with patients without COPD, respectively. Duration of antibiotic treatment during ICU stay was similar in patients with COPD and patients without COPD (11 ± 8 days versus 8 ± 6 days, *p *= 0.224).

### Outcomes of study patients

ICU mortality rate, duration of mechanical ventilation, and duration of ICU stay were significantly higher in cases than in controls and were significantly higher in cases with *S. maltophilia *ICU-acquired infection than in their controls (Table 3).

Although mortality rate was significantly higher in cases with *S. maltophilia *ICU-acquired infection than in cases with *S. maltophilia *ICU-acquired colonisation (21 of 30 [70%] versus 2 of 8 [25%], OR [95% CI] = 2.5 [1.2 to 4.9], *p *= 0.029), duration of mechanical ventilation (22 ± 18 days versus 10 ± 11 days, *p *= 0.104) and duration of ICU stay (29 ± 19 days versus 31 ± 30 days, *p *= 0.847) were similar in the two groups.

In cases with *S. maltophilia *ICU-acquired infection, mortality rate was significantly higher in patients who received inappropriate initial antibiotic treatment than in patients who received appropriate initial antibiotic treatment (8 of 8 [100%] versus 13 of 22 patients [59%], OR [95% CI] = 1.6 [1.1 to 2.3], *p *= 0.035).

### Risk factors for ICU mortality

Risk factors for ICU mortality which wer determined by univariate analysis are presented in Tables 4 and 5. Multivariate analysis identified cardiac failure (OR [95% CI] = 52 [5 to 496], *p *= 0.001), *S. maltophilia *ICU-acquired infection (OR [95% CI] = 2.8 [1 to 7.7], *p *= 0.044), and respiratory failure (OR [95% CI] = 5 [1 to 25], *p *= 0.047) as independent risk factors for ICU mortality.

## Discussion

Our results suggest that *S. maltophilia *colonisation and/or infection is not common in this population of immunocompetent ICU patients. COPD and duration of antibiotic treatment are independently associated with ICU-acquired *S. maltophilia*. ICU-acquired *S. maltophilia *is associated with high ICU mortality and morbidity rates. In addition, ICU-acquired infection related to *S. maltophilia *is an independent risk factor for ICU mortality.

In the SENTRY Antimicrobial Surveillance Program, *S. maltophilia *represented 0.6% to 0.9% of all isolates collected during a three year period [7]. In that study, the respiratory tract was the most commonly reported *S. maltophilia *site of infection in all geographic regions. Incidence of ICU-acquired *S. maltophilia *found by our study (2%) is higher than previously reported. This is probably related to the fact that screening of patients with *S. maltophilia *was not performed in previous studies. Our study differs from previous studies in its methodology. In previous studies, patients with infection related to *S. maltophilia *were compared with patients without *S. maltophilia *infection. However, in our study immunocompetent patients with ICU-acquired *S. maltophilia *were compared with immunocompetent patients without NF-GNB colonisation and/or infection. Risk factors for *S. maltophilia *and other NF-GNB are probably similar. Therefore, exclusion of patients with colonisation and/or infection related to NF-GNB other than *S. maltophilia *probably allowed a more accurate evaluation of risk factors for ICU-acquired *S. maltophilia*.

COPD was identified as an independent risk factor for ICU-acquired *S. maltophilia*. Previous studies identified COPD as a risk factor for VAP and for respiratory tract colonisation by GNB [20,21]. Factors predisposing to respiratory tract colonisation include impairment of mucosal clearance and loss of mucosal integrity [22]. A multicentre study evaluated the relationship between bacterial flora in sputum and functional impairment in patients with acute exacerbation of COPD hospitalised in pneumology units [23]. *P. aeruginosa *was isolated more frequently in patients with low FEV_1 _(forced expiratory volume in one second). Unfortunately, information on severity of COPD was not available in our study. Another potential explanation for the association between COPD and ICU-acquired *S. maltophilia *is the frequent antibiotic use in patients with COPD. Patients with severe COPD are prone to frequent acute exacerbations requiring antimicrobial therapy [24]. In our study, rate of prior antibiotic use was higher in patients with COPD than in patients without COPD. However, this difference did not reach statistical significance.

Prolonged duration of antibiotic treatment is a well-known risk factor for emergence of MDR bacteria [25,26]. Shortening duration of antibiotic treatment could be useful in preventing ICU-acquired *S. maltophilia*. Two recent randomised trials demonstrated that shorter duration of antibiotic treatment is effective and safe in ICU patients with VAP [26,27]. In addition, serial measurements of clinical pulmonary infection score can define the clinical course of VAP resolution [28]. Based on this score, identifying patients with good outcome as early as day three could be of help to define strategies to shorten the duration of antibiotic treatment.

Recent reports documented an increasing incidence of NF-GNB, including *S. maltophilia*, in patients with cystic fibrosis [29,30]. In our study, cystic fibrosis was not significantly associated with *S. maltophilia *acquisition. However, few patients with cystic fibrosis were included in this study. Marchac *et al*. [31] reported that prevalence of *S. maltophilia*, in respiratory tract of patients with cystic fibrosis, increased from 3.3% to 15% during the last decade. Antibiotic treatment, isolation of *Aspergillus fumigatus *in sputum, and oral corticosteroid use were significantly associated with *S. maltophilia *[31]. Another recent study found no association between lung function decline and *S. maltophilia *acquisition in patients with cystic fibrosis, suggesting that *S. maltophilia *is rather a marker of worse lung function in these patients [32].

Carbapenem use was not significantly associated with ICU-acquired *S. maltophilia*. However, previous studies identified carbapenem use as an independent risk factor for *S. maltophilia *acquisition [33,34]. These different results could be explained by the small number of patients treated with carbapenem in our study. However, other studies found no relationship between carbapenem use and *S. maltophilia *acquisition, whereas agents other than carbapenems were identified as risk factors for the acquisition of *S. maltophilia *[35,36]. These findings suggest that exposure to broad-spectrum antibiotics might be more important than exposure to any single agent. Immunosuppression is another well-known risk factor for *S. maltophilia *acquisition [14,37]. Although patients with severe immunosuppression were excluded from our study, ICU patients may be at risk for immunoparalysis. A temporary immunoparalysis may occur in critically ill patients, resulting in higher risk for nosocomial infections [38].

Although risk factors for colonisation and infection related to *S. maltophilia *were similar, outcomes of patients with colonisation or infection related to *S. maltophilia *were clearly different. A recent meta-analysis demonstrated that risk factors for colonisation and infection related to MDR bacteria were similar [39].

In our study, *S. maltophilia *was associated with significantly increased ICU mortality and longer duration of mechanical ventilation and ICU stay. However, *S. maltophilia *was previously considered to have limited pathogenicity. In a recent retrospective study, *S. matophilia *was isolated in the respiratory tract specimens of 64 patients without pneumonia [11]. No significant difference was found in mortality rate between treated and untreated patients. However, few patients (29%) were mechanically ventilated. In another retrospective study, patients with *S. maltophilia *VAP were compared with patients with late-onset VAP related to other GNB [16]. Although mortality rate was similar in patients with *S. maltophilia *VAP and patients with late-onset VAP related to other GNB, *S. maltophilia *was associated with increased patient morbidity. Recent studies indicated that infection with this organism was associated with significant morbidity and mortality, particularly in severely compromised patients [12,13,15]. A retrospective case study, including 69 patients with infection or colonisation related to multi-resistant *S. maltophilia*, has evaluated risk factors for mortality [40]. McCabe score and organ dysfunction were associated with increased risk for mortality in these patients. These results suggest that infection related to *S. maltophilia *has an indirect impact on mortality. In contrast, our results suggest a direct impact of *S. maltophilia *infection on ICU mortality given that this infection was found to be an independent risk factor for ICU mortality. In cases with ICU-acquired infection related to *S. maltophilia*, inappropriate initial antibiotic treatment was associated with increased ICU mortality rate. Inappropriate initial antibiotic treatment is a well-known risk factor for mortality in patients with infection related to NF-GNB [5,6].

Our study has several limitations. First, it was performed in a single ICU. Therefore, our results may not be applicable to other ICU patients. Similarly, only immunocompetent patients were included. This precludes application of our results to patients with immunosuppression. Second, during the study period, no molecular typing was performed on *S. maltophilia *isolates. Therefore, the impact of patient-to-patient transmission could not be determined. Previous studies using molecular typing in newborn and pediatric ICUs have demonstrated that cross-transmission of *S. maltophilia *could be identified using molecular typing [41,42]. However, no *S. maltophilia *outbreak occurred during the study period. Finally, the number of patients with ICU-acquired *S. maltophilia *was small. Therefore, some of the trends observed in this study could have reached statistical significance if the study sample had been larger.

## Conclusion

*S. maltophilia *colonisation and/or infection is not frequent in this cohort of immunocompetent ICU patients. COPD and duration of antibiotic treatment are independently associated with ICU-acquired *S. maltophilia*. ICU-acquired *S. maltophilia *is associated with high mortality and morbidity rates. In addition, ICU-acquired infection related to *S. maltophilia *is an independent risk factor for ICU mortality.

## Key messages

• ICU-acquired *S. maltophilia *is not common among this cohort of immunocompetent ICU patients.

• COPD and duration of antibiotic treatment are independently associated with ICU-acquired *S. maltophilia *and with ICU-acquired infection related to *S. maltophilia*.

• ICU-acquired *S. maltophilia *is associated with high mortality and morbidity rates. In addition, ICU-acquired infection related to *S. maltophilia *is an independent risk factor for ICU mortality.

## Abbreviations

CI = confidence interval; COPD = chronic obstructive pulmonary disease; ICU = intensive care unit; MDR = multi-drug-resistant; NF-GNB = non-fermenting Gram-negative bacilli; OR = odds ratio; SAPS = Simplified Acute Physiology Score; VAP = ventilator-associated pneumonia.

## Competing interests

The authors declare that they have no competing interests.

## Authors' contributions

SN and AD designed the study. All authors contributed to data collection. CDP performed statistical analyses. SN wrote the manuscript, all authors participated in its critical revision. SN had full access to all data in the study and had final response ability for the decision to submit for publication. All authors read and approved the final manuscript.

## Figures and Tables

**Table 1:** Risk factors for ICU-acquired *Stenotrophomonas maltophilia *and ICU-acquired infection related to *S. maltophilia *by univariate analysis

	Cases (*n *= 38)	Controls (*n *= 76)	*p *value	Cases with infection (*n *= 30)	Controls (*n *= 60)	*p *value
At ICU admission						
Age, years	63 ± 12	65 ± 12	0.573	63 ± 13	65 ± 13	0.523
SAPS II	32 ± 15	30 ± 15	0.582	32 ± 15	31 ± 15	0.784
Male gender	24 (63)	50 (65)	0.470	10 (33)	20 (33)	NA
Transfer to the ICU from a ward	31 (81)	65 (85)	0.358	25 (83)	50 (83)	NA
Surgery	9 (23)	18 (23)	NA	7 (23)	14 (23)	NA
Diabetes mellitus	4 (10)	7 (9)	0.532	4 (13)	6 (10)	0.441
COPD	24 (63)	17 (22)	<0.001^a^	20 (66)	12 (20)	<0.001^b^
Cystic fibrosis	3 (7)	2 (2)	0.205	3 (10)	1 (1)	0.106
Prior antibiotic treatment	19 (50)	29 (38)	0.157	15 (50)	23 (38)	0.203
Organ failure						
Respiratory	38 (100)	53 (69)	<0.001^c^	30 (100)	39 (65)	<0.001^d^
Cardiac	8 (18)	13 (17)	0.431	7 (23)	9 (15)	0.267
Renal	9 (23)	16 (21)	0.506	7 (23)	13 (21)	0.561
Neurologic	6 (25)	24 (31)	0.042^e^	4 (13)	20 (33)	0.028^f^
Digestive	1 (2)	8 (10)	0.120	1 (3)	8 (13)	0.119
During ICU hospitalization						
Central venous catheter	35 (92)	69 (90)	0.559	27 (90)	55 (91)	0.537
Urinary catheter	35 (92)	63 (82)	0.147	28 (93)	50 (83)	0.162
Tracheostomy	6 (15)	10 (13)	0.453	4 (13)	6 (10)	0.441
Mechanical ventilation	32 (84)	57 (75)	0.191	30 (100)	57 (95)	0.442
Duration of mechanical ventilation, days	12 ± 10	7 ± 11	0.026	12 ± 10	8 ± 12	0.016
Duration of ICU stay, days	14 ± 11	15 ± 17	0.537	14 ± 12	14 ± 20	0.523

Data are presented as mean ± standard deviation or number (percentage). Odds ratio (95% confidence interval) = ^a^1.9 (1.3 to 2.8), ^b^2.2 (1.3 to 3.5), ^c^1.7 (1.4 to 2), ^d^1.7 (1.4 to 2.1), ^e^2.6 (1 to 7), and ^f^2.4 (1 to 6.2). COPD, chronic obstructive pulmonary disease; ICU, intensive care unit; NA, not applicable; SAPS, Simplified Acute Physiology Score.

**Table 2:** Antibiotic use in study patients during intensive care unit stay

	Cases (*n *= 38)	Controls (*n *= 76)	*p *value	Cases with infection (*n *= 30)	Controls (*n *= 60)	*p *value
Antibiotic treatment	38 (100)	47 (61)	<0.001^a^	30 (100)	36 (60)	<0.001^b^
Duration of antibiotic treatment, days	13 ± 10	5 ± 6	<0.001	16 ± 11	5 ± 6	<0.001
Glycopeptide use	7 (18)	12 (15)	0.457	6 (20)	10 (16)	0.453
Duration, days	1 ± 4	1 ± 2	0.582	2 ± 4	1 ± 2	0.581
Extended-spectrum penicillin use	32 (84)	38 (50)	<0.001^c^	25 (83)	28 (46)	0.001^d^
Duration, days	10 ± 9	4 ± 6	<0.001	11 ± 10	4 ± 6	0.001
Fluoroquinolone use	17 (44)	24 (31)	0.121	12 (40)	17 (28)	0.190
Duration, days	4 ± 6	2 ± 3	0.050	4 ± 6	2 ± 4	0.124
Extended-spectrum cephalosporin use	12 (31)	6 (7)	0.002^e^	9 (30)	5 (83)	0.010^f^
Duration, days	3 ± 6	0.3 ± 1	0.001	4 ± 7	0.3 ± 1	0.004
Carbapenem use	6 (15)	4 (5)	0.067	4 (13)	4 (6)	0.251
Duration, days	4 ± 3	2 ± 1	0.058	1 ± 2	0.4 ± 2	0.294
Aminoglycoside use	11 (28)	14 (18)	0.149	10 (33)	11 (18)	0.095
Duration, days	2 ± 5	1 ± 3	0.154	3 ± 6	1 ± 3	0.080
Metronidazole use	3 (7)	4 (5)	0.429	1 (3)	4 (6)	0.457
Duration, days	0.5 ± 2	0.3 ± 2	0.573	0.3 ± 2	0.4 ± 2	0.532

Results by univariate analysis. Data are presented as mean ± standard deviation or number (percentage). Odds ratio (95% confidence interval) = ^a^1.8 (1.4 to 2.1), ^b^1.8 (1.4 to 2.2), ^c^1.5 (1.2 to 2), ^d^5.7 (1.9 to 16.9), ^e^2.1 (1.1 to 4.2), and ^f^4.7 (1.4 to 15.7).

**Table 3:** Outcomes of study patients

	Cases (*n *= 38)	Controls (*n *= 76)	*p *value	Cases with infection (*n *= 30)	Controls (*n *= 60)	*p *value
Duration of mechanical ventilation, days	23 ± 16	7 ± 11	<0.001	26 ± 17	8 ± 12	<0.001
Duration of ICU stay, days	29 ± 21	15 ± 17	<0.001	29 ± 19	14 ± 20	<0.001
ICU mortality	23 (60)	31 (40)	0.037^a^	21 (70)	23 (38)	0.004^b^

Results by univariate analysis. Data are presented as mean ± standard deviation or number (percentage). Odds ratio (95% confidence interval) = ^a^1.3 (1 to 1.7) and ^b^3.7 (1.4 to 9.5). ICU, intensive care unit.

**Table 4:** Risk factors for ICU mortality by univariate analysis

	Survivors (*n *= 60)	Non-survivors (*n *= 54)	*p *value
At ICU admission			
Age, years	58 ± 19	66 ± 13	0.025
SAPS II	31 ± 16	32 ± 14	0.613
Male gender	36 (60)	38 (70)	0.168
Transfer to the ICU from a ward	47 (78)	49 (91)	0.058
Surgery	10 (17)	17 (31)	0.051
Diabetes mellitus	5 (8)	6 (11)	0.426
COPD	19 (32)	22 (41)	0.208
Cystic fibrosis	3 (5)	2 (4)	0.550
Prior antibiotic treatment	19 (32)	29 (54)	0.014^a^
Organ failure			
Respiratory	42 (70)	49 (91)	0.017^b^
Cardiac	1 (2)	20 (37)	<0.001^c^
Renal	8 (13)	17 (31)	0.024^d^
Neurologic	17 (28)	13 (24)	0.320
Digestive	5 (8)	4 (7)	0.534
During ICU hospitalization			
Central venous catheter	55 (92)	49 (91)	0.560
Urinary catheter	51 (85)	47 (87)	0.484
Tracheostomy	9 (15)	7 (13)	0.484
Mechanical ventilation	43 (72)	46 (85)	0.064
Duration of mechanical ventilation, days	10 ± 14	15 ± 17	0.058
Duration of ICU stay, days	17 ± 19	21 ± 23	0.571
ICU-acquired *Stenotrophomonas maltophilia*	15 (25)	23 (43)	0.037^e^
ICU-acquired infection related to *S. maltophilia*	9 (15)	21 (39)	0.004^f^

Data are presented as mean ± standard deviation or number (percentage). Odds ratio (95% confidence interval) = ^a^2.5 (1.1 to 5.3), ^b^1.48 (1 to 2.1), ^c^3.5 (1.1 to 10.4), ^d^32.9 (4.2 to 256), ^e^2.8 (1 to 7.2), and ^f^3.6 (1.4 to 8.8). COPD, chronic obstructive pulmonary disease; ICU, intensive care unit; SAPS, Simplified Acute Physiology Score.

**Table 5:** Relationship between antibiotic use and intensive care unit mortality in univariate analysis

	Survivors (*n *= 60)	Non-survivors (*n *= 54)	*p *value
Antibiotic treatment	38 (63)	47 (87)	<0.003^a^
Duration of antibiotic treatment, days	7 ± 8	10 ± 10	0.197
Glycopeptide use	9 (15)	11 (20)	0.225
Duration, days	2 ± 3	1 ± 3	0.399
Extended-spectrum penicillin use	34 (56)	38 (70)	0.099
Duration, days	8 ± 8	7 ± 8	0.710
Fluoroquinolone use	22 (36)	24 (44)	0.336
Duration, days	6 ± 6	5 ± 4	0.913
Extended-spectrum cephalosporin use	3 (5)	16 (29)	0.001^b^
Duration, days	1 ± 3	2 ± 5	0.002
Carbapenem use	5 (8)	7 (12)	0.306
Duration, days	3 ± 3	4 ± 1	0.428
Aminoglycoside use	10 (16)	15 (27)	0.233
Duration, days	2 ± 5	2 ± 4	0.154
Metronidazole use	4 (7)	3 (6)	0.559
Duration, days	0.4 ± 2	0.3 ± 2	0.785

Data are presented as mean ± standard deviation or number (percentage). Odds ratio (95% confidence interval) = ^a^3.8 (1.5 to 10) and ^b^7.3 (1.9 to 26.9).
